# Rare ocular manifestations in an 11-year-old girl with incomplete Kawasaki disease: A case report

**DOI:** 10.1097/MD.0000000000010974

**Published:** 2018-06-01

**Authors:** Yunxia Gao, Yifan Zhang, Fang Lu, Xiaoyue Wang, Ming Zhang

**Affiliations:** a Department of Ophthalmology, Sichuan University West China Hospital, Chengdu, Sichuan; b Sichuan University West China School of Medicine, China.

**Keywords:** Kawasaki disease, ocular manifestations, retinal detachment, retinal vasculitis

## Abstract

**Introduction:**

Kawasaki disease is a necrotizing vasculitis featuring fever, erythema, conjunctivitis, and lymphadenopathy. Ocular manifestations in Kawasaki disease are commonly limited to anterior segment, posterior segment lesions are rarely reported.

**Case presentation:**

We report a unique case of ocular manifestations in an 11-year-old girl with incomplete Kawasaki disease. An 11-year-old Asian girl presented with severe enophthalmos, retinitis, retinal detachment, and choroidal detachment secondary to an unexplained fever for 10 days.

**Conclusion:**

To the best of our knowledge, this is the first documented case of incomplete Kawasaki disease with severe posterior segment lesions. The local use of dexamethasone in the eye was effective in our patient. Surgical intervention might not be necessary even though the initial symptoms could be devastating. The eye should be monitoring the eye routinely in patients with Kawasaki disease.

## 1 Introduction

Kawasaki disease is a necrotizing vasculitis featuring fever, erythema, conjunctivitis, and lymphadenopathy.^[1]^ Ocular manifestations in Kawasaki disease are commonly limited to anterior segment characterized by bilateral bulbar conjunctivitis without exudate, superficial punctate keratitis, uveitis, or vitreous opacities.^[2]^

We found 6 literatures^[3–7]^ describing posterior segment lesions in Kawasaki disease. But none of them progressed to retinal detachment. Only one literature^[7]^ described a patient lost vision in 1 eye due to retinal and vitreous exudation. We report a unique case of incomplete Kawasaki disease (atypical) with severe enophthalmos, cataracts, exudative retinal detachment, and retinal vasculitis.

## 2 Report of a case

An 11-year-old Asian girl presented with a 10-day history of fever (up to 40.5 °C), generalized rash, erythema of oral mucosa, cervical lymphadenopathy, bilateral bulbar conjunctivitis without exudate, and cervical lymphadenopathy. Laboratory results showed elevated white blood cell count with neutrophil predominance C-reaction protein, erythrocyte sedimentation rate, plantlet count, serum liver enzymes, and decreased albumin. Patient did not have strawberry tongue, erythema, and edema of the hands and feet. Electrocardiogram showed no abnormal findings. Incomplete (atypical) Kawasaki disease was suspected (Table 1).^[1,8–11]^

**Table 1:**
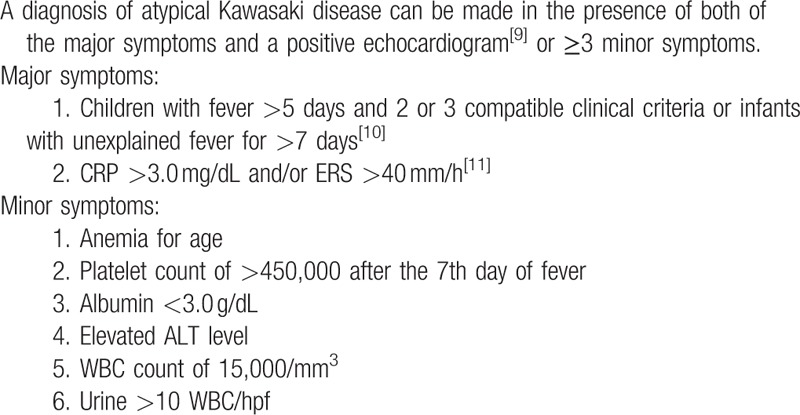
Diagnosis of atypical Kawasaki disease^[1,8]^.

Patient was immediately treated with intravenous immunoglobulin (IVIG) for 10 days in a community hospital. Unfortunately, she responded poorly to the treatment, the fever persisted despite the infusion of immunoglobulin. Ten days later, she was transferred to our university hospital. We added high-dose corticosteroids to her treatment regimen.

**Figure 1:**
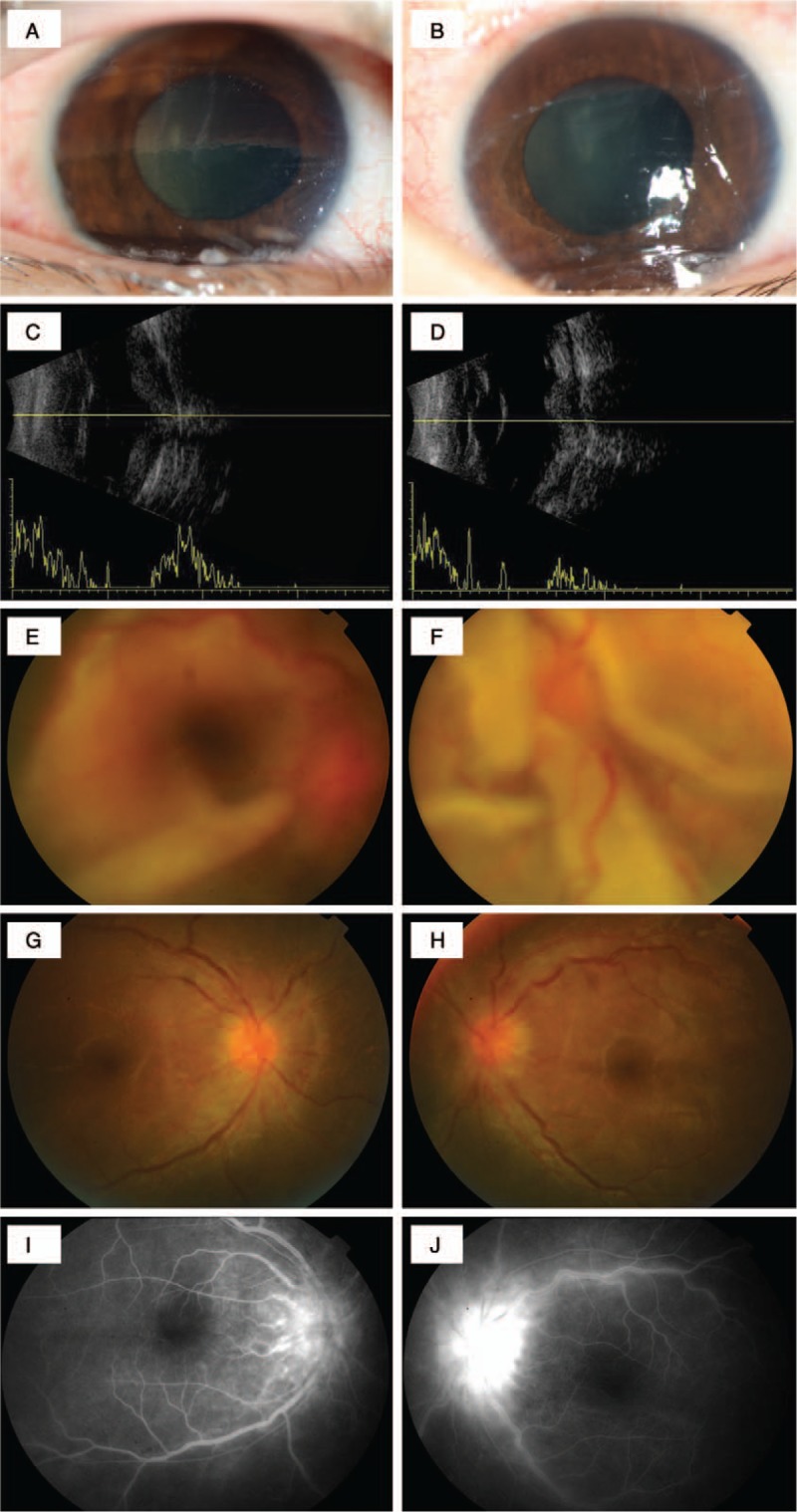
A, Photograph of the right eye, and B, photograph of the left eye showed severe deformation, conjunctival edema, and injection without exudate, corneal edema with folds. C, B-scan of the right eye, and D, B-scan of the left eye, revealed shortened ocular axis, bilateral retinochoroidal folds. E, Funduscopic photograph of the right eye, and F, funduscopic photograph of the left eye, indicated retinal detachment, choroidal detachment, and extensive retinochoroidal folds. G, Funduscopic photograph at 2-week of the right eye and, H, funduscopic photograph at 2-week follow-up of the left eye demonstrated optic disk swelling and flattened retinas. I, Fluorescein fundus angiography at 2-week follow up of the right eye, and J, fluorescein fundus angiography at 2-week follow up of the left eye showed hyperfluorescence of the disc and fluorescein leakage.

Two days after we started her on intravenous dexamethasone (30 mg/d for 4 days and 15 mg/d for 5 days), the patient was referred to the ophthalmology clinic presenting with ophthalmodynia, photophobia, and enophthalmos. Visual acuity was HM/5 cm OU. Ocular B-scan showed severe reduction of ocular volume and bilateral chorioretinal folds. Axial length was 15.6 mm OD and 16.1 mm OS (Fig. 1C and D). Intraocular pressure (IOP) was extremely low in OU (unmeasurable). Ophthalmology examination showed conjunctival edema and injection without exudate, corneal edema with folds (Fig. 1A and B), lens opacity, 3+ aqueous flare, and 1+ keratin precipitates.

Fundus photography suggested retinal detachment, choroidal detachment, and extensive chorioretinal folds (Fig. 1E and D). A fluorescein fundus angiography was obtained 2 weeks later when the ocular inflammation slightly resolved, which revealed hyperfluorescence of the disc and fluorescein leakage (Fig. 1I and J).

**Figure 2:**
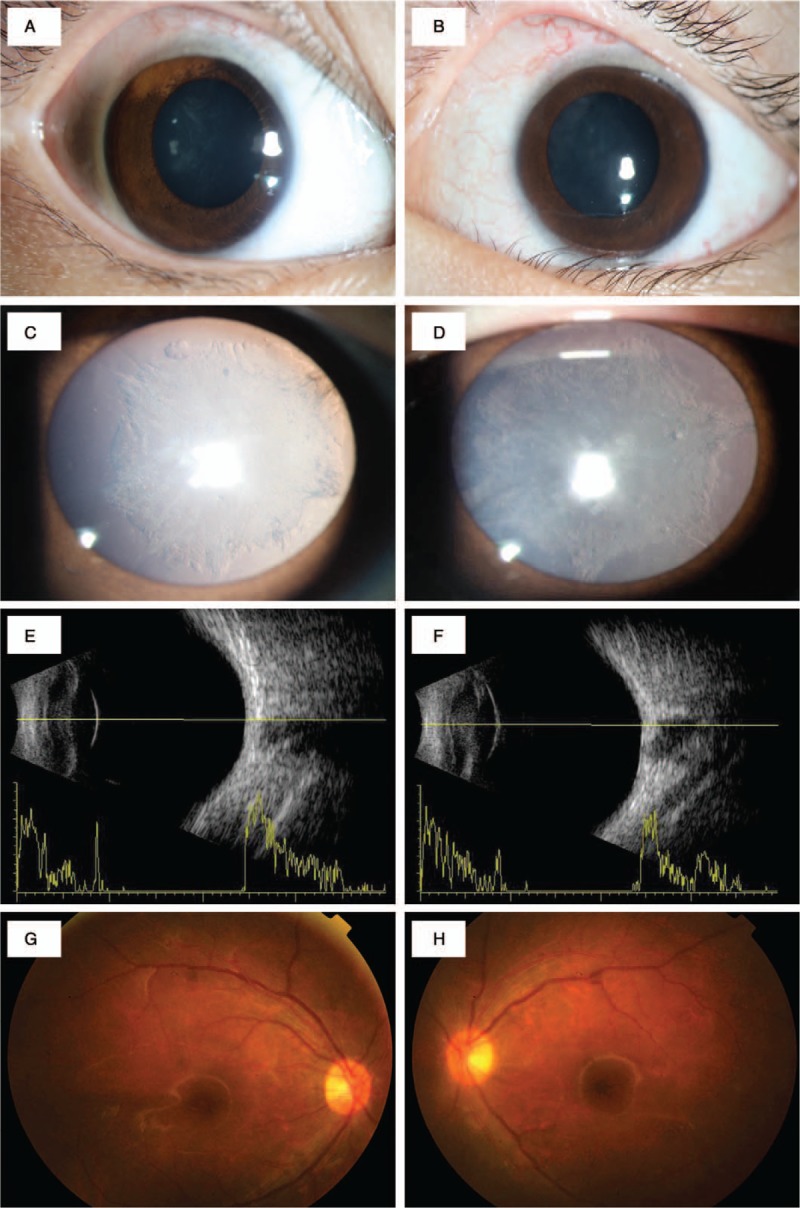
A and B, Photographs at 2-month follow-up suggested inflammation resolved. Both eyes looked normal. C and D, photographs of the anterior segment at 2-month follow-up showed posterior subcapsular opacities. E and F, B-scans at 2-month follow-up reveled normal axial length and normal retinas. G and H, Funduscopic photographs at 2-month follow-up showed normal retinas.

Tobramycin dexamethasone eye drops and atropine eye drops were given. The fever and rash started to resolved gradually 4 days after the administration of dexamethasone. She was discharged after 15 days of hospitalization. At follow-ups, retinas gradually flattened and axial length recovered to normal. At 2-month follow-up, visual acuity recovered to 20/50 OU. IOP was normal in OU. Fundus photography revealed normal retinas (Fig. 2G and H). Ophthalmology examination showed posterior subcapsular opacities and normal retinas (Fig. 2C and D). At 24-month follow-up, visual acuity recovered to 20/25 OU (Fig. 2).

## 3 Discussion

To the best of our knowledge, this is the first documented case of incomplete Kawasaki disease with severe reduction of ocular volume, cataracts, retinal vasculitis, and retinal detachment. We report this case wishing to raise the awareness of posterior segment involvement, especially retinal lesions in Kawasaki disease.

Our patient showed 2 features. She is an older child (10 years old) who didn’t present with all the typical symptoms of Kawasaki disease. Despite the correct diagnosis and treatment, her fever was poorly controlled. She had resistance to intravenous immunoglobulin. The devastating enophthalmos, ocular hypotony, and retinal detachment could very likely be the result of the prolonged course of systemic inflammation.

We learned 3 lessons from our practice. The local use of dexamethasone in the eye was effective in our patient. Surgical intervention might not be necessary, given the ocular inflammation could resolve itself as the systemic condition improves. Unnecessary surgeries might cause further damage. Whether the patient needs a surgery depends largely on the evaluation and judgment of the ophthalmologist. Monitoring the eye and the fundus routinely, while treating Kawasaki disease systemically, benefits patients with retinal lesions,^[4]^ and potentially prevents the ocular inflammation progress to vision loss.

## Acknowledgment

The authors thank Mr. Dan Meng and Mr. Yongzhi Huang from Imaging Department, Sichuan University West China Hospital for the excellent work on fundus photography and fluorescein fundus angiography.

## Author contributions

**Conceptualization:** Yunxia Gao, Ming Zhang.

**Data curation:** Yunxia Gao, Yifan Zhang, Xiaoyue Wang, Ming Zhang.

**Formal analysis:** Yunxia Gao, Yifan Zhang, Fang Lu.

**Investigation:** Yunxia Gao, Fang Lu, Ming Zhang.

**Methodology:** Fang Lu.

**Project administration:** Yifan Zhang, Ming Zhang.

**Resources:** Yunxia Gao, Xiaoyue Wang, Ming Zhang.

**Supervision:** Yunxia Gao, Fang Lu, Ming Zhang.

**Writing – original draft:** Yifan Zhang.

**Writing – review and editing:** Yunxia Gao, Yifan Zhang, Ming Zhang.
